# Removal of Ethanol from Indoor Air by *Ficus elastica* Roxb.: Process Optimisation and Post-Removal Desorption Dynamics

**DOI:** 10.3390/plants15162484

**Published:** 2026-08-16

**Authors:** Abayhan Buran, Aykut Topdemir

**Affiliations:** Bioengineering Department, Faculty of Engineering, Fırat University, Elazığ 23119, Türkiye; atopdemir@firat.edu.tr

**Keywords:** indoor air quality, ethanol removal, botanical filtration, *Ficus elastica*, response surface methodology

## Abstract

Indoor air pollution caused by volatile organic compounds (VOCs) is a major environmental and public health concern. Ethanol is a common indoor VOC released from cleaning products, disinfectants, and industrial activities. This study evaluated the capacity of *Ficus elastica* Roxb. to remove airborne ethanol under controlled chamber conditions. Response Surface Methodology was applied to optimise the effects of initial ethanol concentration, relative humidity, and exposure time on removal efficiency. The model predicted a maximum removal efficiency of 97.16%, which was experimentally validated with an average efficiency of 96.2%, confirming the model’s reliability. Analysis of variance identified exposure time as the most influential factor affecting ethanol removal. Desorption experiments showed only limited and transient ethanol re-emission, indicating that ethanol was not merely adsorbed but also partially metabolised by the plant. Scanning electron microscopy revealed structural changes in stomatal morphology after prolonged exposure. Biochemical analyses demonstrated increased total phenolic content, flavonoid content, and antioxidant capacity, whereas a moderate decline in total chlorophyll reflected physiological stress accompanied by enhanced defence responses, indicating adaptive tolerance to prolonged ethanol exposure. These findings demonstrate the potential of *F. elastica* as an effective and sustainable botanical biofiltration system for improving indoor air quality.

## 1. Introduction

Volatile organic compounds (VOCs) are a group of various organic chemicals characterised by their ability to evaporate at room temperature and are therefore predominantly found in the atmosphere as gases. These compounds are significant due to their widespread occurrence and potential adverse effects on human health and the environment. VOCs are emitted from various sources, including natural sources, industrial processes, vehicle emissions, household products, and vegetation [1,2].

Indoors, VOCs can be emitted from numerous sources, including paints, cleaning agents, adhesives, and building materials [3,4]. The accumulation of these compounds can lead to a condition known as ‘sick building syndrome,’ where building occupants experience health problems directly linked to indoor air quality [5,6]. Studies have shown that prolonged exposure to VOCs can exacerbate asthma and allergy symptoms, particularly in sensitive populations such as children and individuals with pre-existing health conditions [3,6].

VOCs are known to cause a range of health problems, primarily respiratory issues. Studies have shown that exposure to VOCs can lead to decreased lung function, particularly in sensitive populations such as the elderly [7,8]. For example, one study showed that elderly individuals exposed to indoor VOCs experienced higher rates of shortness of breath and respiratory failure [8]. Furthermore, VOCs can exacerbate conditions such as asthma and allergic reactions because they cause inflammation and irritation of the respiratory tract [9,10]. The presence of VOCs in enclosed spaces has been associated with acute effects such as headaches, dizziness, and irritation of the eyes, nose, and throat [8,11].

With the COVID-19 pandemic, the increased use of disinfectants and antiseptic solutions has led to a sudden rise in the level of ethanol vapour exposure. Ethanol, which is highly volatile, is known to have neurotoxic effects, particularly with chronic exposure. It can impair cognitive function and cause neurological symptoms such as headaches and dizziness [12]. Occupational exposure guidelines provide an additional reference point for the concentrations tested here: the NIOSH/OSHA permissible exposure limit and ACGIH threshold limit value for ethanol are all set at 1000 ppm as an 8 h time-weighted average, with an IDLH (immediately dangerous to life or health) threshold of 3300 ppm [13]. Studies show that ethanol vapour concentrations can cause acute health effects. For example, exposure to an ethanol vapour concentration of approximately 1900 mg/m^3^ for three hours caused slight increases in blood ethanol levels, but these remained below 2 mg/L, suggesting that even relatively high concentrations may have measurable effects on human physiology [14]. The potential for ethanol to act as a central nervous system depressant raises concerns regarding its inhalation in enclosed spaces, particularly environments where individuals may be exposed for extended periods, such as homes or offices. Furthermore, the effect of ethanol vapours on microbial growth in enclosed spaces has been documented; this suggests that while ethanol may inhibit certain pathogens, it may also contribute to the growth of others, potentially leading to an imbalance in indoor microbial communities [15]. This imbalance could exacerbate health issues such as respiratory infections or allergic reactions, particularly in sensitive individuals.

Botanical biofiltration has emerged as a promising method for removing volatile organic compounds from indoor air. This technique utilises the natural processes of plants and associated microbial communities to degrade or absorb pollutants, thereby improving indoor air quality. Research indicates that microorganisms in the rhizosphere play a crucial role in VOC degradation and typically account for a significant portion of removal efficiency [16,17]. The design of botanical biofilters significantly influences their effectiveness in VOC removal. Active green walls and vertical plant garden systems have been developed to maximise plant surface area and increase air exposure to the root zone [18,19]. These systems facilitate forced airflow throughout the plant growing medium, increasing contact between air and plants, which in turn increases VOC removal rates. Research has shown that such systems can achieve higher removal efficiency, particularly for indoor air pollutants, compared to traditional potted plants [18,20].

The mechanisms by which plants remove ethanol involve both physical absorption and metabolic conversion. Ethanol can be absorbed through the roots and then metabolised in plant tissues, where it can be converted into less harmful compounds or utilised in various metabolic pathways [21]. For example, the presence of ethanol has been shown to increase the removal efficiency of other pollutants in biotrap filters inoculated with certain fungal species and to indicate a synergistic effect [22]. This suggests that the interaction between ethanol and microbial communities associated with plant roots can significantly affect the overall removal efficiency of VOCs.

*Ficus elastica* has large, thick, leathery leaves covered by a well-developed waxy cuticle, providing an extensive lipophilic surface for VOC adsorption in addition to stomatal uptake [23], while its high total leaf area per plant maximises the air-contact surface available for removal. It is one of the most widely cultivated houseplants in homes and offices worldwide. It has independently demonstrated substantial removal capacity for common indoor VOCs, such as benzene (up to 93.86% [24]) and toluene (up to 92.01% [25]) under comparable closed-chamber conditions. It is also low-maintenance and tolerant of the variable light and low-ventilation conditions typical of indoor spaces, making it both practically relevant and well-suited to prolonged chamber exposure.

In this study, a botanical air filtration system based on *F. elastica* Roxb. was investigated for the removal of ethanol from indoor air. The removal process was optimised using Response Surface Methodology by evaluating the combined effects of ethanol concentration, relative humidity, and exposure time on removal efficiency. In addition to process optimisation, the potential for post-removal ethanol desorption, changes in leaf stomatal morphology, and alterations in bioactive compounds induced by ethanol exposure were comprehensively evaluated. We hypothesised that *F. elastica* can efficiently remove ethanol from indoor air under optimised environmental conditions, while exhibiting minimal post-removal desorption. Furthermore, we hypothesised that prolonged ethanol exposure would induce measurable physiological and biochemical responses, reflected by changes in stomatal morphology, photosynthetic pigments, phenolic compounds, flavonoids, and antioxidant capacity. This study therefore aimed to optimise ethanol removal efficiency while providing a comprehensive evaluation of the structural and biochemical responses of *F. elastica* to ethanol exposure.

## 2. Results

A series of test procedures were implemented with the objective of predicting the loss percentages and adhesion that may occur on the chamber following ethanol injection. In order to achieve this objective, 1000 ppm ethanol was vaporised within the chamber. Air samples were collected from the chamber at 24 and 48 h and analysed using gas chromatography (GC). The ethanol concentrations were calculated as percentages and are shown in Table 1.

In experiments conducted solely with pots and test plants, the entire pot (so that the soil surface was not visible) was covered with aluminium foil. During the experimental procedure, a substantial increase in the loss of ethanol was observed. This increase was 82.0%, and it was observed at a temperature of 25 °C under ambient conditions. The experiment was conducted with a potted test plant placed in the cabinet, without any variables being adjusted. This demonstrated that the above-ground parts of the plant played an active role in the removal of ethanol, due to the absence of a connection between the soil and the indoor air. A series of experiments were conducted in order to ascertain whether the plant itself was capable of removing VOCs from the indoor environment, without giving consideration to factors such as temperature, light, and humidity.

### 2.1. Optimisation of Ethanol Removal from Indoor Air Using F. elastica

RSM optimisation was performed using the Design Expert 10 programme to determine the optimum values of the ethanol concentration that the *F. elastica* plant can remove from the indoor environment, taking into account different variables. The variables selected for ethanol optimisation were VOC concentration (ppm), relative humidity (%) and time (hours).

The model created for the ethanol removal efficiency of the *F. elastica* plant was comprehensively evaluated in terms of analysis of variance (ANOVA), model fit, and statistical criteria (Table 2). The variance analysis concluded that the model was overall highly significant (*p* < 0.0001) and demonstrated a high level of explanatory power (F = 330.81).

Interactions between factors (AB, AC, BC) were not significant, indicating that individual factors were primarily effective in the system. All of the model’s quadratic components (A^2^, B^2^, C^2^) were found to be highly significant (*p* < 0.0001), and the non-linear effect of time was particularly strong. Statistical evaluations of the model’s fit also yielded very positive results. The R^2^ value was calculated as 0.9967, the adjusted R^2^ value as 0.9936, and the predictive R^2^ value as 0.9743. The proximity of these values demonstrates the model’s high explanatory and predictive power (Table 3). The model’s coefficient of variation (CV) is 1.20%, indicating the consistency of the experimental data, while the adequate precision value of 60.499 points to a very high signal-to-noise ratio.

Of the three primary factors, duration (C) had the most significant effect on ethanol removal and was found to be highly significant (F = 2209.07; *p* < 0.0001). The variables concentration (A) and relative humidity (B) were also found to be statistically significant with values of F = 71.69 (*p* < 0.0001) and F = 17.66 (*p* = 0.0018), respectively.

**Figure 1:**
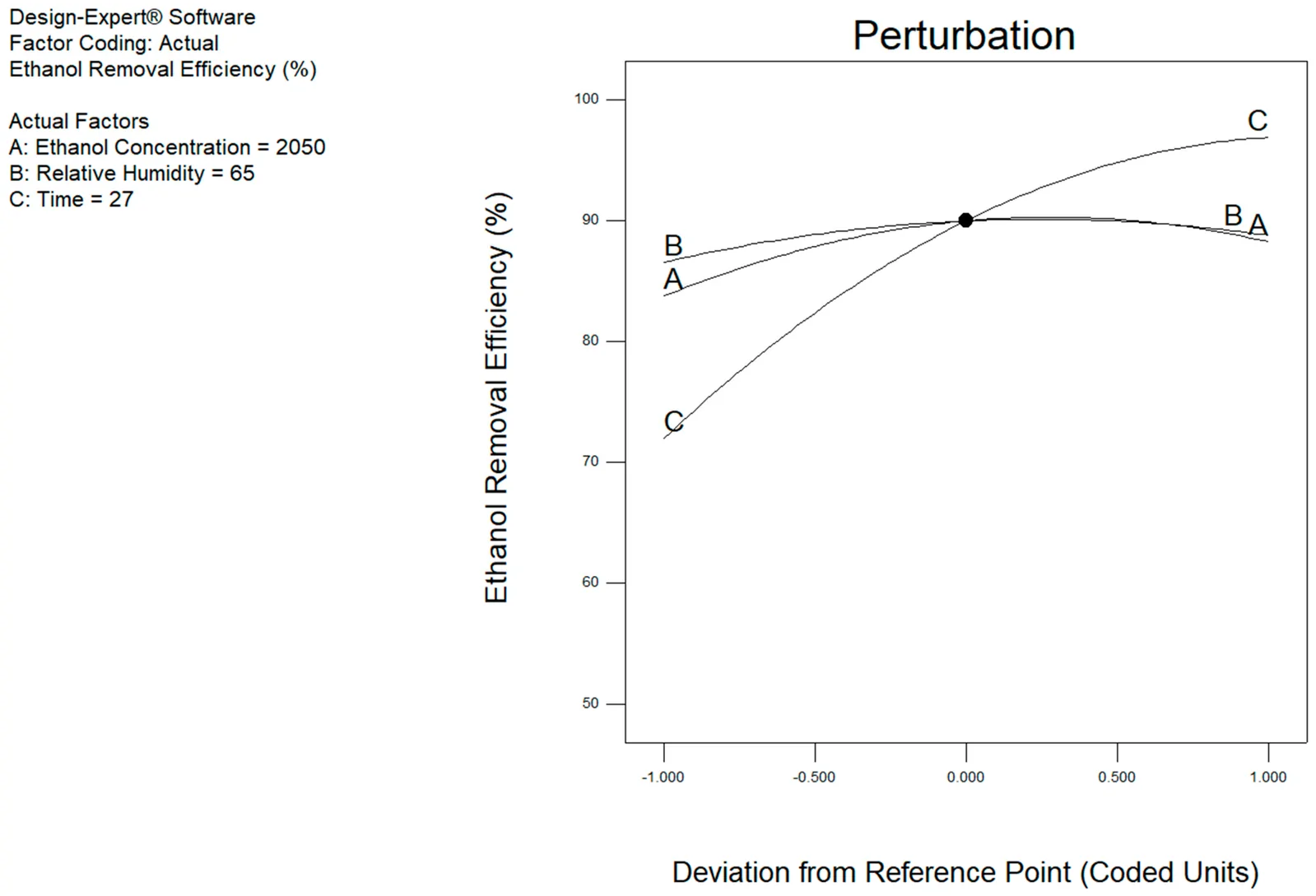
A comparison of variables is to be made based on deviation from the specified reference point.

As demonstrated in Figure 1, it is evident that when each factor is modified individually from a reference point where all factors are constant, there is a corresponding alteration in the response variable (ethanol removal percentage).

In the resulting perturbation graph, the C factor (time) is characterised by the steepest curve and is identified as the parameter with the most significant effect on ethanol removal. A positive increase in time has been shown to significantly enhance removal efficiency, a finding that is consistent with the high F value (F = 2209.07) and significant *p*-value (*p* < 0.0001) for the C factor in the model’s ANOVA.

**Figure 2:**
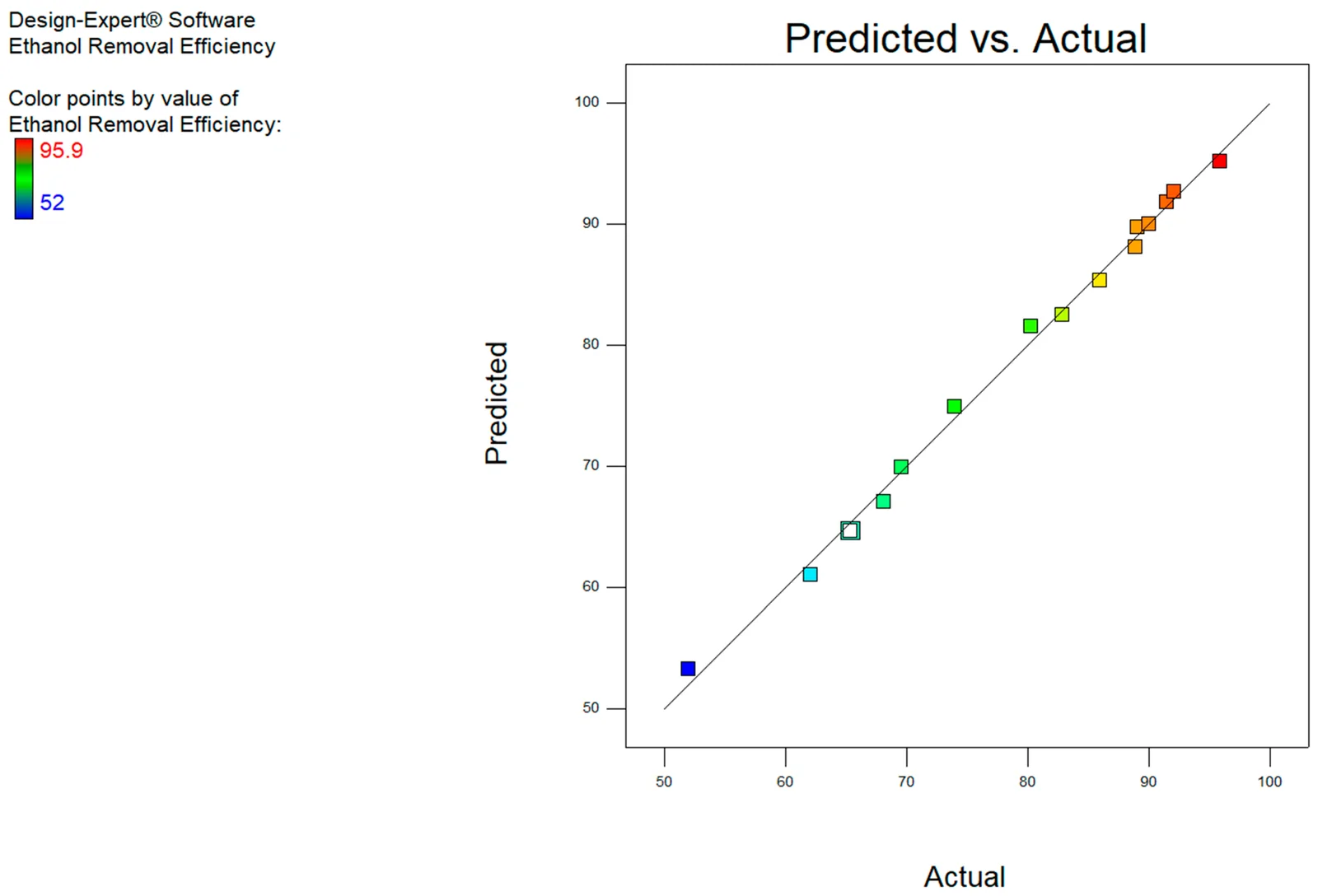
Predicted vs. actual graph created to assess the accuracy and validity level of the model.

Figure 2 compares the ethanol removal percentage predicted by the model with the actual values obtained through experimental means.

In an ideal regression model, it is expected that all data points will lie on or very close to the line of best fit y = x. This linear relationship indicates that the model produces highly accurate predictions. The observation that the data points are in close proximity to the linear line also suggests that the model successfully represents non-linear effects and interactions and is not prone to significant overfitting. Moreover, the absence of any outliers suggests that the experimental design was consistent.

**Figure 3:**
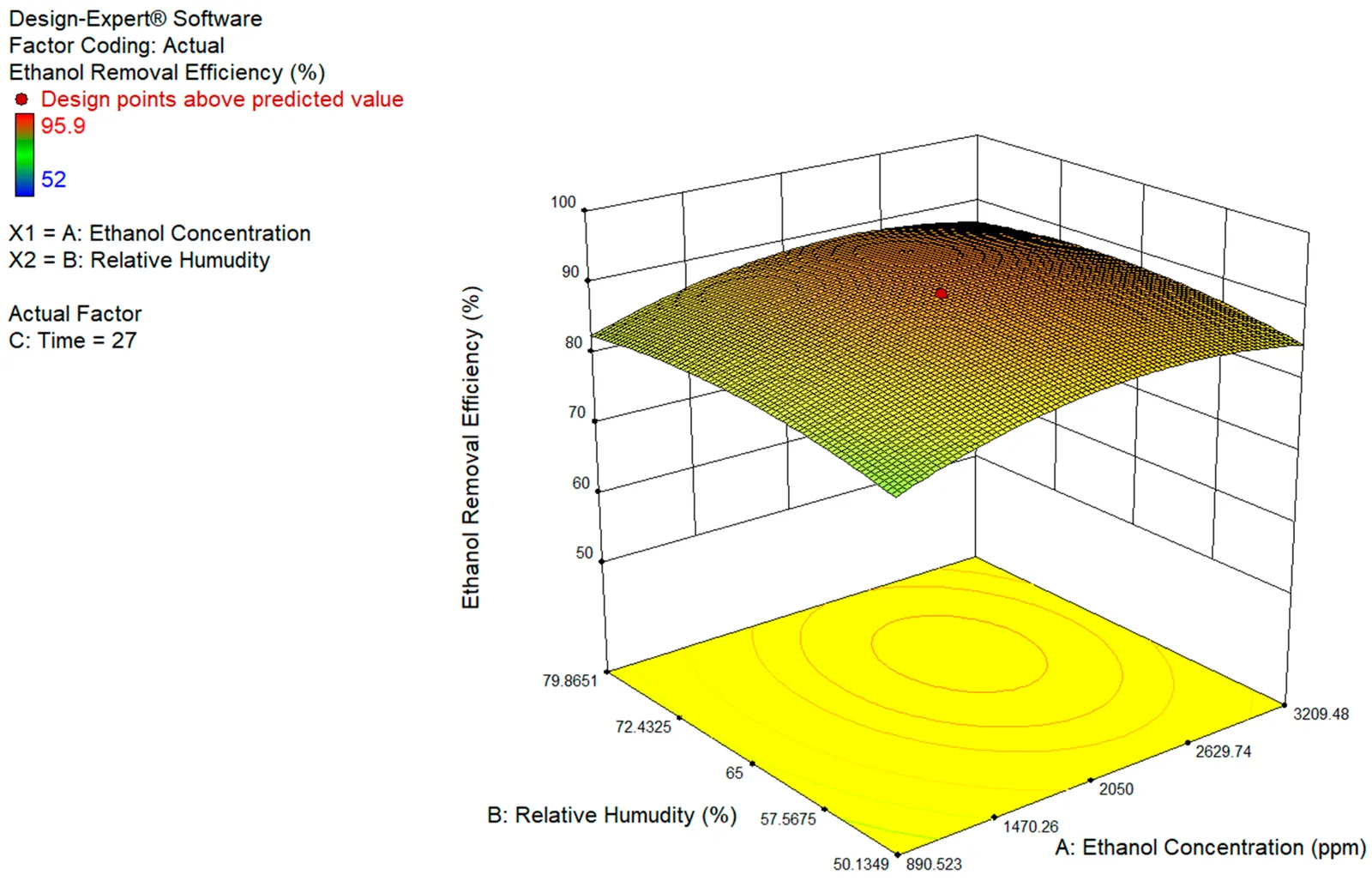
Three-dimensional surface graph illustrates the combined effect of ethanol concentration (A) and relative humidity (B) variables on the ethanol removal percentage under constant time conditions (C = 27 h) on the efficiency of the process.

The three-dimensional response surface graph in Figure 3 demonstrates that the ethanol removal efficiency under experimental conditions increases with both the ethanol concentration and relative humidity parameters, reaching a maximum at a certain point.

The graph’s dome-like structure indicates the presence of an area where both factors are optimised in conjunction, while the sections where the surface begins to flatten suggest that the optimum has been exceeded or that the saturation point is imminent. When the ethanol concentration is approximately 2050 ppm and the relative humidity is around 65%, it has been observed that the ethanol removal percentage approaches optimal values (approximately 95%). This finding is consistent with the results of the variance analysis, which indicated that both factors were significant individually (*p* < 0.0001). Conversely, the slope of the surface is predominantly horizontal in the direction of moisture, suggesting that the relative effect of the moisture factor is more constrained.

**Figure 4:**
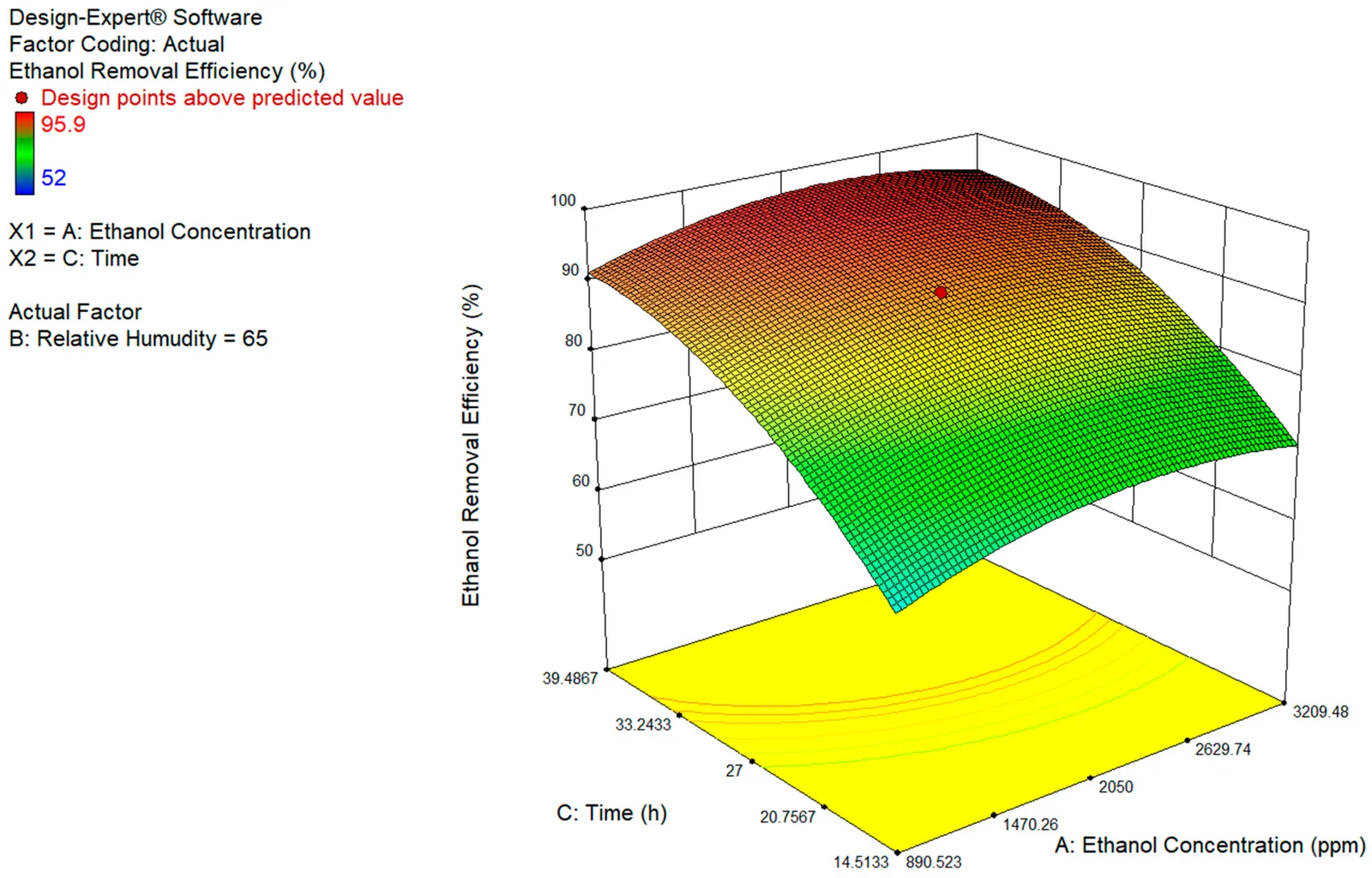
Three-dimensional surface graph illustrates the impact of the variables “Ethanol Concentration” (A) and “Time” (C) on the ethanol removal percentage (B) under constant humidity (B = 65%) conditions on the cost-effectiveness of the process.

As demonstrated in Figure 4, an increase in both variables results in a substantial enhancement of VOC removal efficiency. In particular, the C factor (time) is the axis where the curvature of the surface is more pronounced, and it can be seen that the effect of this variable on the response variable is higher.

This finding is consistent with the C factor demonstrating the highest F value (2209.07) in the ANOVA. The highest yield regions on the surface are located in the upper right part of the graph, and these demonstrate that a removal efficiency of nearly 95% is achieved under conditions of approximately 3200 ppm VOC concentration and nearly 40 h duration.

**Figure 5:**
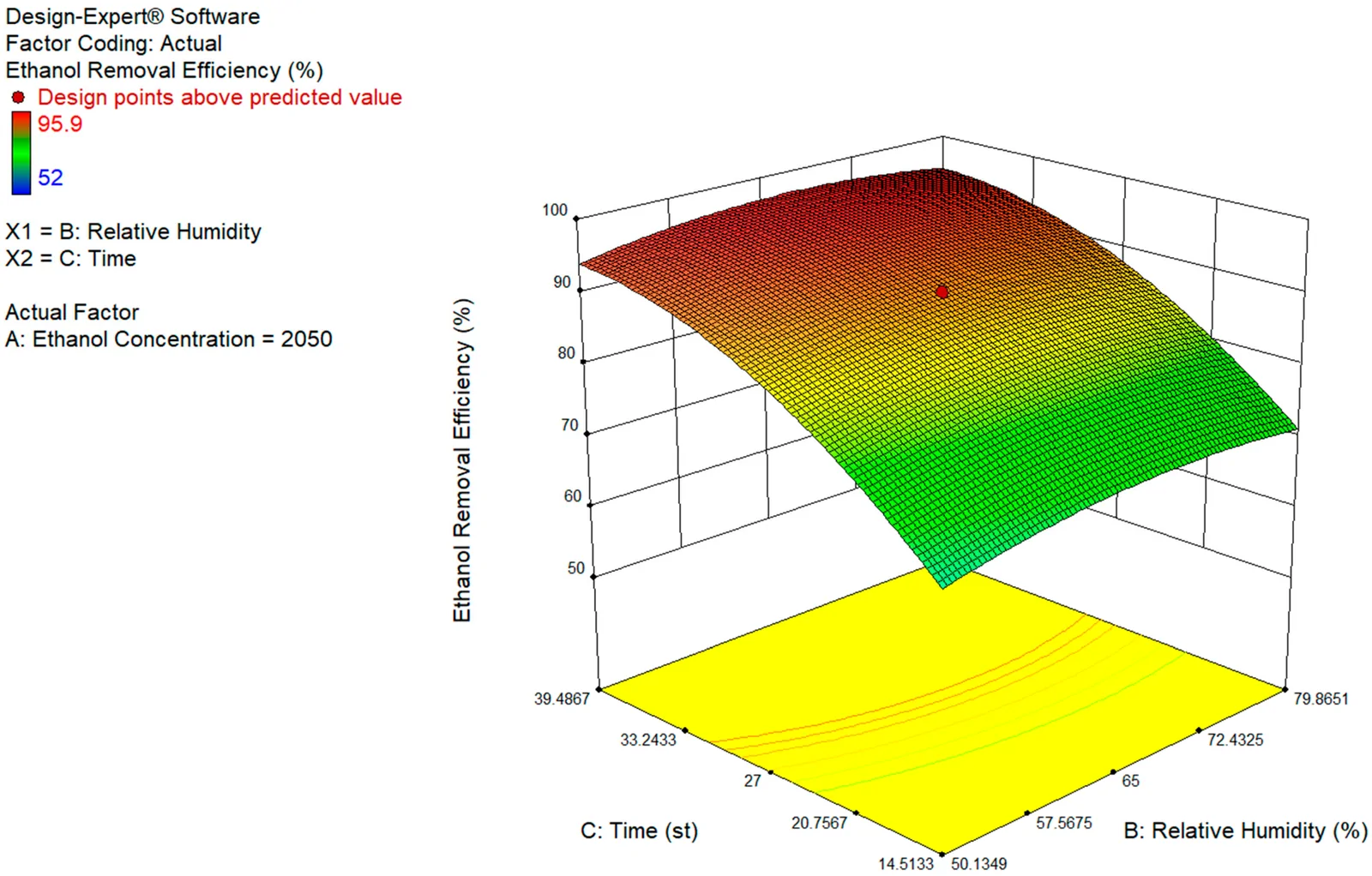
Three-dimensional surface graph illustrates the combined effects of relative humidity (B) and time (C) variables on the percentage of ethanol removed at a VOC concentration (A = 2050 ppm) that is below the removal efficiency.

As demonstrated in Figure 5, the steeper gradient on the time axis is indicative of a more pronounced effect on the removal efficiency over time. As time progresses, there is a marked increase in the efficiency of ethanol removal, suggesting that mechanisms such as adsorption or biological degradation become increasingly significant over time.

This hypothesis is corroborated by the results of the analysis of variance (ANOVA), whereby the C factor (time) demonstrates a high F value (2209.07) and a significant *p*-value (*p* < 0.0001).

**Figure 6:**
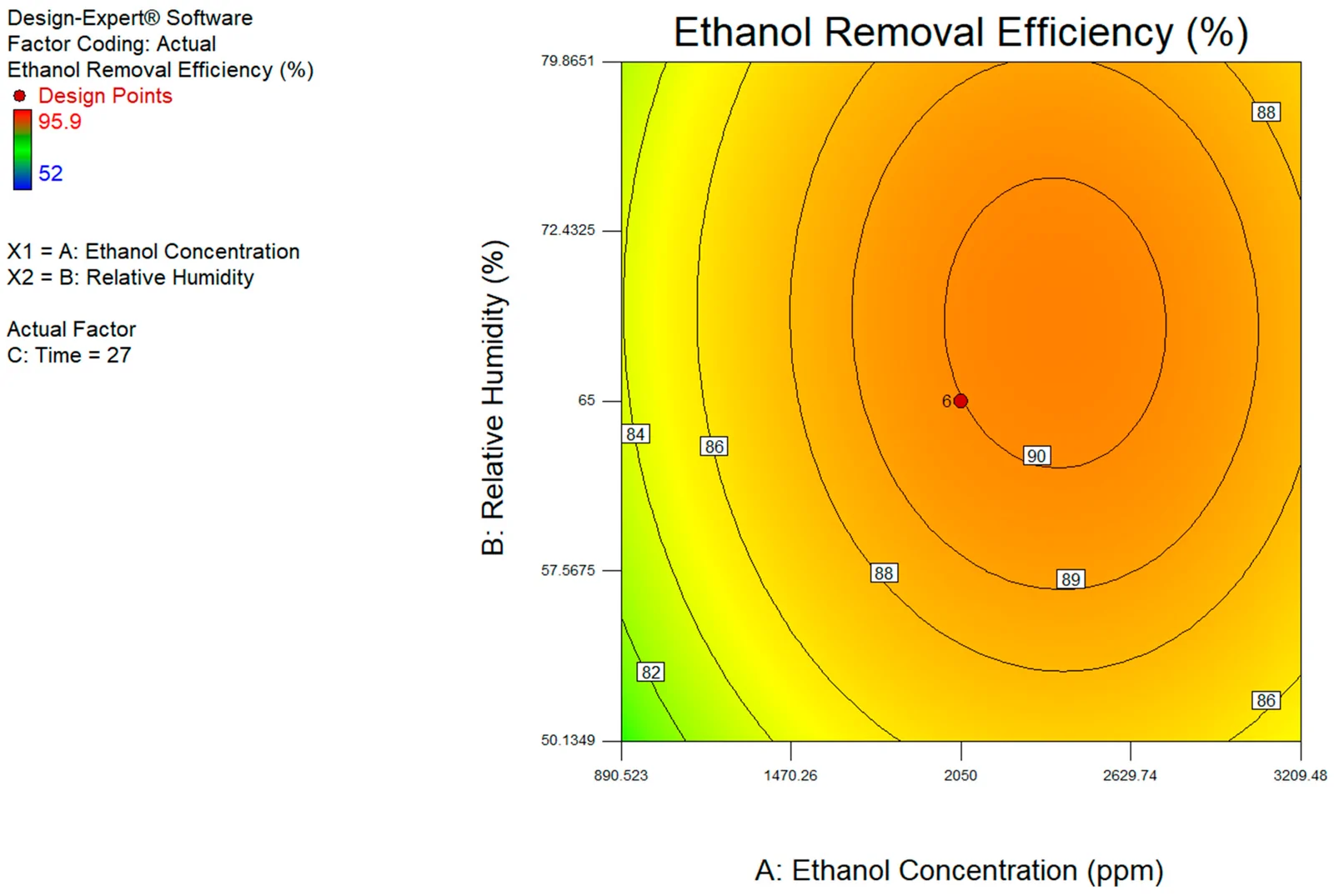
A contour plot visually representing the effects of ethanol concentration (A) and relative humidity (B) on yield efficiency under a constant duration condition (C = 27 h) in two dimensions.

As illustrated in Figure 6, the distribution of the ethanol removal percentage predicted by the model is represented on a two-dimensional plane. The utilisation of colour transitions and isocontours serves to visually represent the anticipated removal efficiency at different relative humidity and ethanol concentration values. The darker orange regions in proximity to the centre of the graph denote areas where ethanol removal attains its maximum values.

A salient feature of the graph is the observation that the removal percentage increases concomitantly with both the increase in ethanol concentration and the rise in relative humidity. In particular, removal rates reach values of 90% and above at an ethanol concentration of approximately 2050 ppm and a relative humidity level of 65–70%. This region is indicative of the target values in terms of optimisation.

Upon examination of the slope of the isocon lines, it is understood that they are denser and more closely spaced along the ethanol concentration axis. This finding indicates that the effect of concentration on removal efficiency is more pronounced. A more gradual slope is observed along the relative humidity axis, indicating that humidity also exerts a significant effect (*p* = 0.0018 in ANOVA), albeit to a lesser extent than concentration.

### 2.2. Maximum Ethanol Removal Solutions Achieved Through the Optimisation Process

The results obtained from the optimisation of ethanol removal efficiency are presented in Table 4. These results are based on the maximisation of the ethanol removal percentage, under the condition that the ethanol concentration, relative humidity and time variables are kept within the specified value ranges. Consequently, the maximum ethanol removal efficiency was determined to be 97.160%.

As illustrated in Table 4, the most efficacious solution combinations, obtained through the implementation of RSM optimisation techniques with the objective of maximising ethanol removal efficiency, have been identified. The response variable, VOC Removal Efficiency, was considered, and the desirability function reached a high value of 0.941. This finding suggests that, within the confines of the model’s specified parameters, the target response has been optimised and the solution is statistically reliable. The maximum ethanol removal yield was calculated to be 97.160%, and four solutions were proposed to achieve this value. In these solutions, the VOC concentration ranges from approximately 2276–2282 ppm, the relative humidity is around 66.7–66.8%, and the application time is 40.8 h.

As illustrated in Table 5, the data were obtained from five independent replicate experiments, all conducted under the same fixed optimal conditions (ethanol concentration, relative humidity, and exposure time) identified by the RSM model, in order to confirm the reproducibility of the predicted maximum ethanol removal efficiency. The ethanol removal percentage obtained in each experiment ranged from 95.7 to 96.7%, with an average of 96.2% calculated for the experiments.

The findings indicate that the optimisation estimates derived from mathematical modelling have been empirically substantiated with a high degree of accuracy. It is acknowledged that minor discrepancies may be observed between experiments, and these can be attributed to the inherent variability in biological material. This finding is significant in terms of the sustainability and reproducibility of the optimisation and supports the reliability of the model.

### 2.3. Ethanol Removal Under Optimal Conditions Using Micropropagated Plants

The results of serial experiments conducted with new plants produced through micropropagation, which were used to determine the ethanol removal efficiency, are presented in Table 6. Removal efficiency was assessed using a single test unit consisting of two micropropagated *F. elastica* plants co-cultivated in one pot (combined total leaf area: 0.81 m^2^). Utilising genetically identical plants, cultivated under in vitro conditions, devoid of any contaminants, and exhibiting equivalent environmental and adaptation characteristics, ensured that only variable parameters could be focused on, as a single type of plant was employed in the analyses and optimisation. The findings from the validation experiments conducted with micropropagated plants demonstrate that the ethanol removal percentage exhibited a gradual increase as the experiment sequence number increased. This phenomenon can be interpreted as an adaptive response capacity and system stabilisation of the plant system, despite the optimal conditions (concentration, relative humidity, duration) determined during the optimisation process being maintained constant. The enhancement in the extraction rate throughout the series of experiments signifies that the micropropagated plants underwent a gradual acclimatisation process to the environment to which they were exposed, and that metabolic activity stabilised over time and commenced functioning at an optimal level.

### 2.4. Desorption Tests

**Figure 7:**
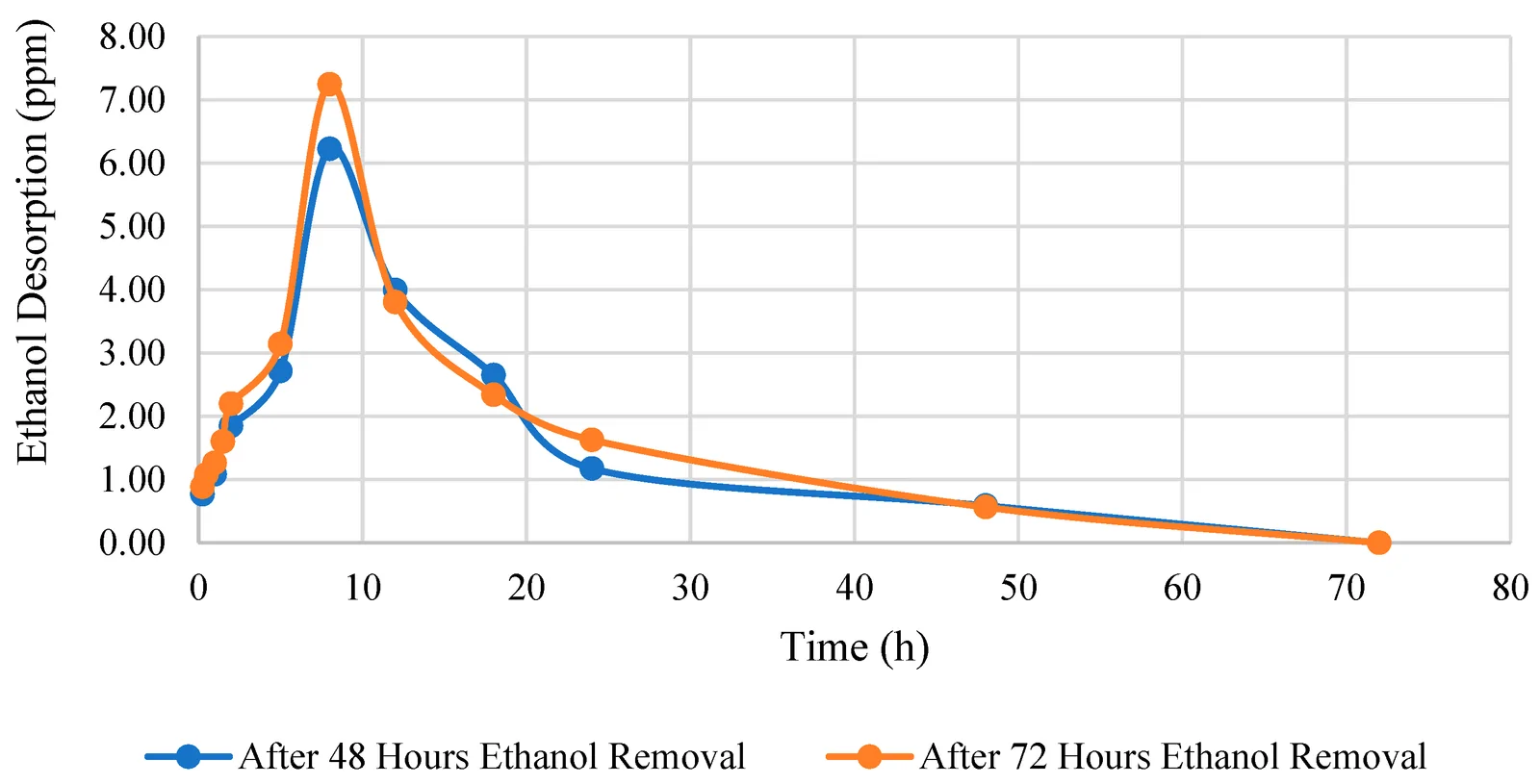
Comparative graph of desorption concentrations (ethanol) obtained based on the periods determined following the 48 h and 72 h removal tests.

Desorption analyses indicated minor and short-lived re-emission of ethanol, implying that the compound was not merely physically retained but was at least partially subjected to metabolic transformation within the plant–microbial system. As demonstrated in Figure 7, a comparative graph is provided of the desorption concentrations obtained after 48 and 72 h of removal. This increase was found to be 0.48 ppm. As demonstrated by both sets of test results, there was a rapid decrease in the ethanol concentration in the environment at the conclusion of the ninth hour. The results of this study indicate that *F. elastica* possesses the capacity to retain volatile organic compounds within its structure for a designated period of time, subsequently releasing these compounds to a limited extent. However, this release is observed to remain at low levels in terms of environmental risk. The minimal ethanol desorption observed indicates that it is efficiently metabolised by plant or soil microbiota.

It has been hypothesised that certain species of plants may release volatile organic compounds at minimal levels in uncontaminated environments subsequent to the absorption of these compounds from the surrounding environment. Nevertheless, this does not pose a threat to environmental health. This phenomenon can be particularly evident in cases where plants are unable to effectively metabolise or store VOCs following their absorption. This phenomenon is typically transient and manifests in minimal quantities. In order to reduce the risk of desorption, the utilisation of plant species that are capable of metabolising VOCs is recommended. It may be more effective for the plant to be exposed to a polluted environment on a regular basis, in a manner analogous to a purification agent.

#### 2.4.1. SEM Images of *F. elastica* Explants

**Figure 8:**
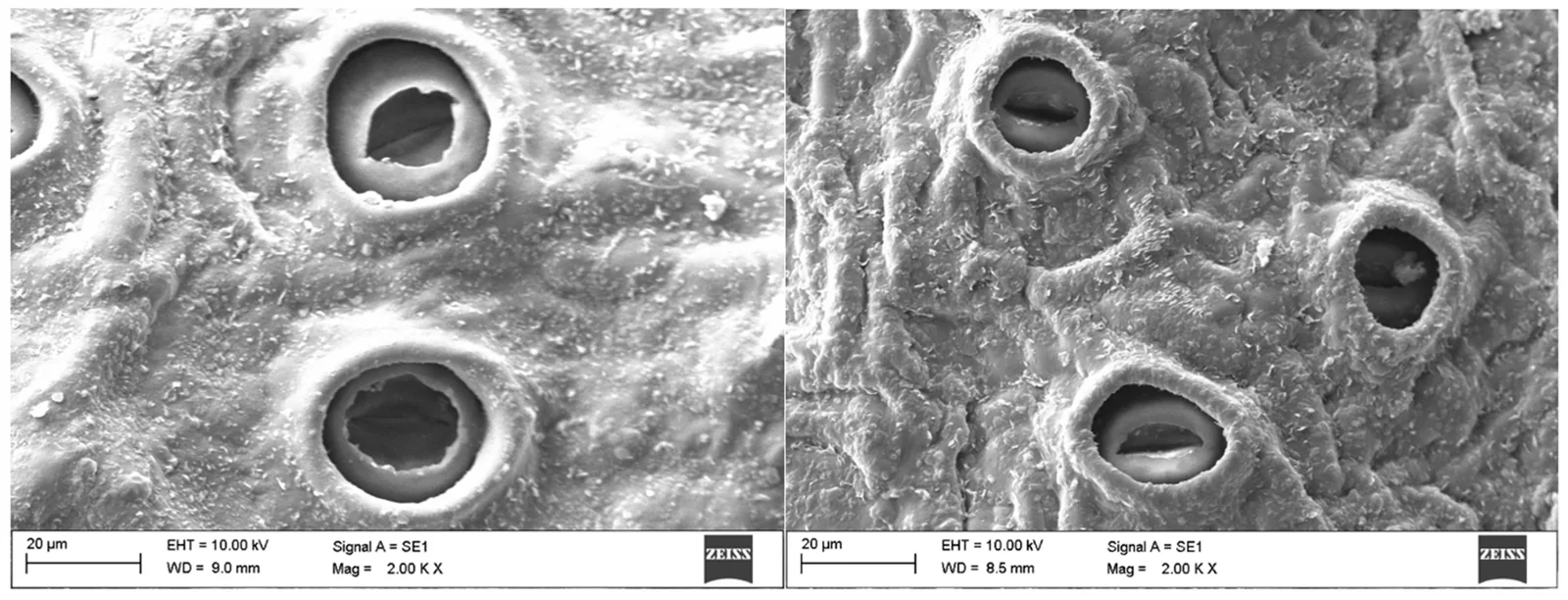
SEM image showing tissue damage in stomatal structures and changes in the cuticle layer after 120 h of ethanol exposure (**left**) and SEM image of healthy stomatal structures and cuticle structure in a leaf sample taken from an unexposed plant (**right**).

Surface changes have been observed in leaves exposed to ethanol. It was found that there was partial deformation around the stomata in samples that had been exposed to 2000 ppm ethanol for 120 h. The presence of granular particles and microscopic residues was observed on the surface (Figure 8). The release of extracellular metabolites or cuticular changes caused by stress may be the underlying cause of these particles. Despite the absence of a substantial decline in stoma frequency, the stomata exhibited reduced aperture width. This finding suggests that the plant may have adopted a protective strategy against VOC stress by diminishing gas exchange.

A visual comparison of the stoma structures in samples from unexposed plant not exposed to ethanol with those from samples exposed to ethanol reveals clear visibility of the stoma structures and a smooth appearance of the surrounding tissues. In addition, no lipid residue-like irregular particles or surface roughness are observed between the stomata. This finding lends further support to the hypothesis that exposure to VOCs exerts deleterious effects on leaf surface anatomy, potentially resulting in morphological alterations to cuticle–stoma structures.

**Figure 9:**
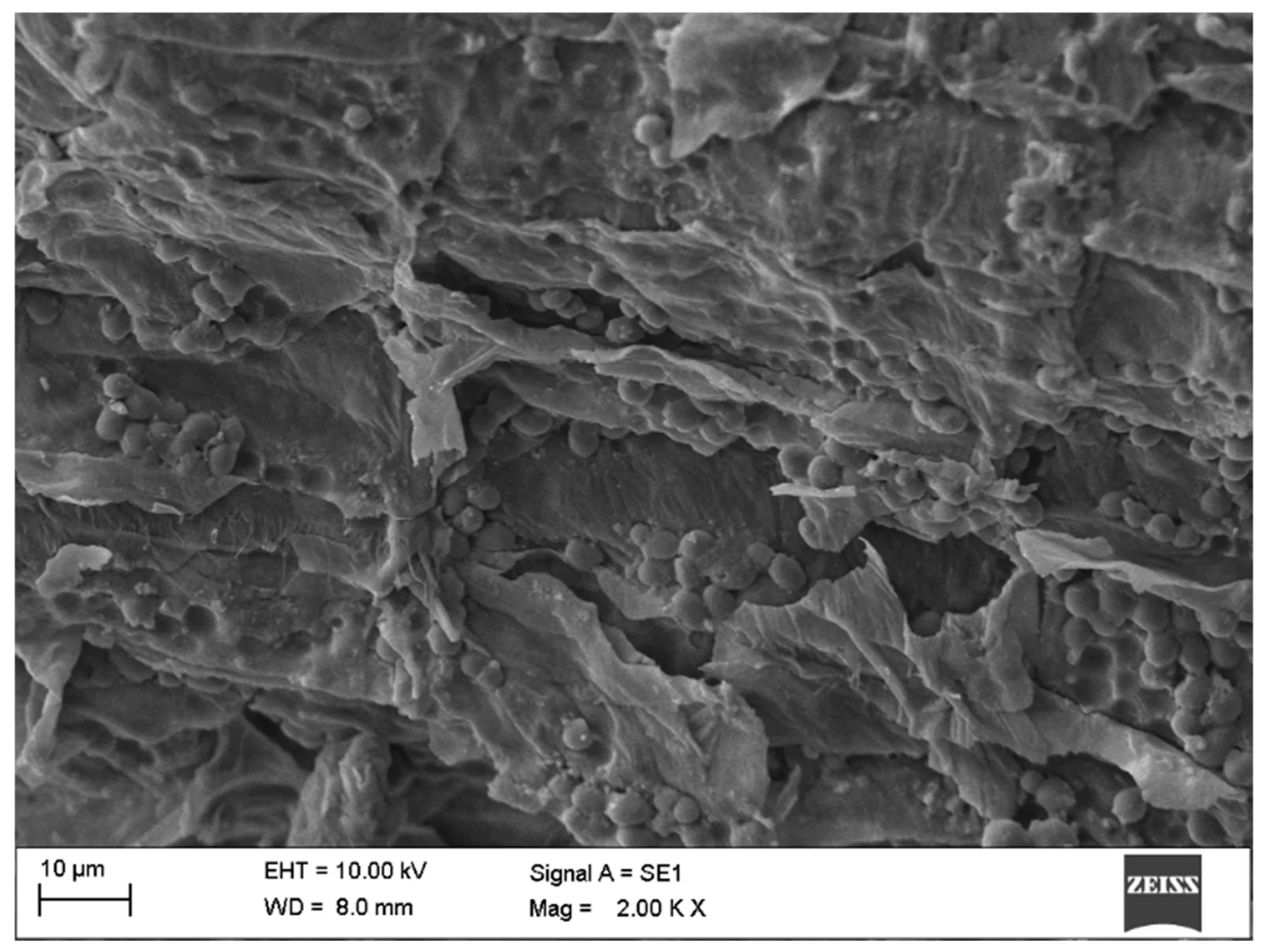
SEM image at 2 KX magnification of the root explant obtained after the optimisation process.

At 2000× magnification in the root explant (Figure 9), dense granular accumulations in the form of spherical structures are observed in the intercellular spaces and around the cell walls. The morphology and disposition of these granules are consistent with polysaccharide or starch accumulations resulting from the plant’s metabolic defence mechanism. Indeed, it is well-documented that plants accumulate reserve substances, such as starch, with a view to preventing carbon waste and storing energy under stress conditions [26]. Concurrently, these accumulations may act as a defence against oxidative stress triggered by the penetration of compounds such as ethanol into cell walls.

#### 2.4.2. Amounts of Bioactive Components

The total chlorophyll, chlorophyll-a, chlorophyll-b, and total carotenoid content in *F. elastica* leaves were determined following the long-term exposure (120 h ethanol—120 E) experiments conducted in this study. The objective of this study is to observe and interpret the changes in bioactive substance content resulting from the tests performed. Furthermore, the total phenolic content, total flavonoid content, and total antioxidant capacity of the leaf explants were determined through analyses. Consequently, the leaf explants obtained after the tests were compared with extracts from unexposed plant leaves that had not been exposed to any VOCs (Table 7, Table 8, Table 9 and Table 10).

As shown in Table 7, leaves exposed to ethanol for 120 h (120 E) exhibited higher total phenolic content than unexposed shoots across all solvent systems. In both tissue types, the highest phenolic concentrations were obtained using ethyl acetate (120 E: 58.14 mg GAE; unexposed plant: 52.98 mg GAE), followed by ethanol, while distilled water yielded the lowest values. The consistently elevated phenolic levels in 120 E samples suggest that prolonged ethanol exposure stimulated phenolic biosynthesis. This increase likely reflects activation of plant defence mechanisms under sustained stress conditions.

As shown in Table 8, total flavonoid content was also higher in leaves subjected to 120 h of ethanol exposure compared to unexposed plants. Ethyl acetate extracts yielded the highest flavonoid concentrations in both 120 E (64.38 mg QE) and unexposed plant samples (60.06 mg QE), whereas distilled water extracts produced the lowest values. The pronounced increase observed in organic solvent extracts of 120 E leaves indicates that prolonged ethanol exposure may enhance flavonoid biosynthesis. This elevation can be interpreted as an adaptive response to oxidative stress induced by ethanol treatment.

As shown in Table 9, total antioxidant capacity (TEAC) followed a trend consistent with phenolic and flavonoid contents. Leaves exposed to ethanol for 120 h demonstrated higher antioxidant capacity than unexposed plants in all solvent systems. The highest TEAC values were recorded in ethyl acetate extracts (120 E: 9.23 mmol/g TEAC; unexposed plant: 8.83 mmol/g TEAC), while distilled water extracts showed comparatively lower activity. These findings suggest that ethanol-induced stress activated antioxidant defence mechanisms, likely associated with increased accumulation of phenolic and flavonoid compounds.

As shown in Table 10, photosynthetic pigment content displayed a pattern distinct from that of secondary metabolites. Unexposed plants contained higher levels of total chlorophyll, chlorophyll-a, and chlorophyll-b compared to leaves exposed to ethanol for 120 h. In contrast, total carotenoid content was slightly higher in the 120 E samples. These results indicate that prolonged ethanol exposure may suppress photosynthetic pigment accumulation while promoting or maintaining protective carotenoid levels. Overall, 120 h ethanol exposure appears to enhance secondary metabolite production while exerting a potentially inhibitory effect on photosynthetic capacity.

The decrease in chlorophyll content suggests that prolonged ethanol exposure may have impaired the photosynthetic apparatus through oxidative stress. In contrast, the increased phenolic and flavonoid contents indicate activation of antioxidant defence mechanisms, representing a metabolic shift from primary to secondary metabolism. The observed reduction in chlorophyll may also reflect nitrogen reallocation from photosynthetic pigment synthesis to the production of stress-related metabolites. Although ethanol removal efficiency remained high during the experimental period, prolonged exposure may adversely affect long-term plant performance and the sustainability of continuous VOC removal.

## 3. Discussion

It has been demonstrated that higher initial VOC concentrations can lead to increased diffusion rates, thereby enabling greater removal by plants [27]. This finding is consistent with the results of the present study, in which higher concentrations yielded a greater percentage of removal compared to lower initial concentrations. However, if the concentration is excessively high, it may exceed the plant’s capacity to process these compounds effectively, leading to potential phytotoxic effects [28]. For this reason, RSM was applied to determine the optimal concentration range and to quantitatively evaluate the influence of both the lower and upper concentration limits, together with relative humidity and exposure time, on ethanol removal efficiency.

The RSM model developed in this study explained 99.67% of the variance in ethanol removal efficiency (R^2^ = 0.9967, F = 330.81, *p* < 0.0001), and the close agreement between the predicted maximum (97.16%) and the experimentally validated value (96.2%) confirms strong predictive reliability. This level of fit compares favourably with RSM-based optimisation studies of other biological VOC removal systems: for example, an RSM model of a biofilter treating toluene achieved a markedly lower R^2^ (0.8826) using the same modelling framework [29], suggesting that the closed-chamber design used in the present study reduced the environmental noise that typically limits RSM performance in less controlled biofiltration systems.

Among the three variables, exposure time exerted by far the strongest effect (F = 2209.07), exceeding both ethanol concentration (F = 71.69) and relative humidity (F = 17.66), and none of the two-factor interactions reached significance. This indicates that pollutant uptake by *F. elastica* is predominantly a time-dependent biological process rather than an instantaneous adsorption event, consistent with Thomas and Kays [30], who reported that VOC removal efficiency in phytoremediation systems increases markedly with contact time. A recent modelling study of botanical biofiltration [31] similarly frames VOC removal as the cumulative outcome of several concurrently operating pathways—cuticular/stomatal uptake, microbial degradation in the rhizosphere [16,17], root-zone diffusion [27], and phytodegradation within plant tissue—each of which accumulates its effect over time rather than acting as a single-step partitioning event. This framework helps explain both the dominance of time in the present model and the lack of significant interaction terms, suggesting these mechanisms act largely in parallel rather than synergistically within the tested range.

The maximum efficiency obtained here (97.16% predicted, 96.2% validated, at ~2282 ppm ethanol, 66.7% RH, 40.8 h) is high relative to efficiencies reported for VOC removal by other botanical systems, several of which report lower or more variable performance depending on ventilation and system design [20,32,33]. It is also comparable to, or exceeds, removal levels previously reported for *F. elastica* against other VOCs: a BTX mixture reduced to 4.0 ± 0.5 µL/L within 12 h [34], and formaldehyde reduced from 1.9–2.2 mg/m^3^ to 1.27 mg/m^3^ [35]. Given that ethanol is smaller and considerably more water-soluble than aromatic hydrocarbons such as toluene or benzene, its higher aqueous solubility may facilitate more efficient partitioning into moist stomatal cavities and cell walls, which could partly explain the high removal percentages obtained here relative to less water-soluble VOCs.

At the same time, this efficiency was obtained under sealed, static-air chamber conditions without continuous air exchange—a condition that does not reflect real indoor environments. Cummings and Waring [32] specifically caution that potted-plant VOC removal reported under sealed-chamber conditions tends to overestimate real-room performance once realistic air-exchange rates are accounted for.

Relative humidity had a smaller, though still significant, effect on removal efficiency than time or concentration (F = 17.66, *p* = 0.0018), with an optimum around 66–67% RH. This partly agrees with Huang et al. [36], who showed that relative humidity affects the gas–surface partitioning behaviour of VOCs and therefore their availability for uptake, and with Cho et al. [37], who reported that excessive humidity can promote competitive adsorption between water vapour and VOC molecules at limited binding sites. However, the comparatively moderate influence of humidity observed here—well below the effect of time—diverges somewhat from studies in which humidity was reported as a stronger driver of VOC uptake; this may reflect ethanol’s high water miscibility, making its uptake less sensitive to competitive water-vapour effects than more hydrophobic VOCs. As Cummings and Waring [32] note, the direction and magnitude of humidity effects on plant-mediated VOC removal remain inconsistent across the literature, and the present findings add a data point specific to ethanol and *F. elastica*. A similar result was observed in a separate experiment involving the removal of toluene using *F. elastica*. While the VOC contact time was the most critical factor, no significant effect of relative humidity was observed [25]. The solubility of toluene and ethanol in water is also a key factor that must be considered.

Under constant optimal conditions, ethanol removal efficiency in the micropropagated-plant validation series rose steadily across ten consecutive runs (93.5% to 95.9%) rather than remaining flat. Because these plants were genetically identical and grown under controlled in vitro conditions before testing, this trend more plausibly reflects physiological acclimatisation than genetic variability. Micropropagated plantlets are well documented to undergo a distinct adjustment phase when moved from sterile in vitro conditions to ex vitro environments, during which stomatal functioning, cuticle development, and antioxidant defences progressively stabilise [38]. A parallel study tracking antioxidant metabolism during ex vitro acclimatisation of *Stevia rebaudiana* found that oxidative stress indicators peaked in the first days and then progressively resolved as protective enzymatic pathways were activated over the following one to two weeks [39]—a pattern conceptually consistent with the gradual improvement in removal performance observed here. An additional, non-exclusive explanation is a possible build-up of rhizosphere or phyllosphere microbial communities over repeated ethanol exposures, which could contribute an increasing microbial-degradation component across sequential runs [16,17].

Desorption tests showed only a small increase in ethanol concentration (0.48 ppm) between the 48 and 72 h trials, with a sharp decline after approximately nine hours, indicating that ethanol removal by *F. elastica* is largely irreversible under the tested conditions. This contrasts with purely physisorption-based technologies: reviews of VOC removal by physical adsorbents such as activated carbon and metal–organic frameworks describe desorption of more weakly bound compounds under competitive or thermal conditions as a recognised limitation of these media [40]. The degree of affinity exhibited by different VOCs for plant tissues and microbial communities in the soil is subject to variation [41]. For instance, some VOCs may be more readily absorbed or metabolised by certain plant species, resulting in differences in degradation rates. Furthermore, the interaction between VOCs and microbial communities in the rhizosphere is crucial, as these microorganisms are often responsible for a significant portion of VOC degradation [16,42]. The low reversibility observed here is more consistent with a biologically active system in which uptake, microbial degradation, and phytodegradation act together to transform pollutants rather than merely trap them at a surface [31,43], supporting the interpretation, already noted in the Results section, that ethanol was partially metabolised rather than simply retained.

SEM imaging after 120 h of exposure to 2000 ppm ethanol revealed a reduction in stomatal aperture width without a corresponding decline in stomatal frequency, together with granular starch-like accumulations in root tissue. A recent diffusion-resistance model of VOC uptake by plant leaves identified the metabolic conversion step, rather than the stomatal pathway itself, as typically the most limiting resistance in the overall absorption pathway, with stomatal and plasma-membrane resistance ranking second depending on the compound [44]. Within this framework, the stress-induced narrowing of stomatal aperture observed here would still be expected to reduce the stomatal component of VOC entry, and is consistent with stomatal closure being a documented defensive response to reactive-gas exposure more broadly—water-stressed Brassica nigra plants, for instance, reduced ozone uptake specifically through stomatal closure [45]. This raises a practical implication: under very prolonged or high-concentration exposure, the same protective narrowing that limits gas influx may also constrain the stomatal contribution to VOC removal, even though metabolic capacity—not stomatal aperture alone—appears to be the primary bottleneck for overall uptake. The starch-like granular accumulation in root tissue is consistent with the carbon-storage and stress-defence role described by Fahad et al. [26], reinforcing that both above- and below-ground tissues mount coordinated metabolic responses to sustained ethanol exposure.

After 120 h of ethanol exposure, *F. elastica* leaves showed higher total phenolic content, flavonoid content, and antioxidant capacity than unexposed young shoots, alongside a moderate reduction in total chlorophyll and a slight increase in total carotenoids. This pattern closely mirrors findings from a recent study, in which exogenous ethanol treatment of three herbaceous species increased radical-scavenging activity and total phenolic content while reducing chlorophyll and carotenoid levels at higher ethanol concentrations, consistent with ethanol-induced oxidative stress and a phenylpropanoid-mediated antioxidant defence response [46]. The convergence between that hydroponic (root-zone) ethanol-exposure study and the present airborne-exposure system suggests that this stress-defence signature may be a relatively general plant response to ethanol, regardless of exposure route. The slight increase in carotenoids despite falling chlorophyll is consistent with carotenoids’ photoprotective role and their tendency to be maintained even as the main photosynthetic pigment pool is compromised. Several limitations should be acknowledged. First, all optimisation and validation experiments were conducted in sealed chambers without continuous air exchange, which likely overestimates removal efficiency attainable in ventilated, occupied spaces [32]; future work should evaluate performance under realistic air-exchange rates. Second, the long-term physiological and biochemical assessments were based on a single prolonged exposure regime (2000 ppm, 120 h) in one plant batch, so the generalisability of the stress-response magnitude to other concentrations, durations, or plant ages remains to be established. Third, this study did not directly quantify the relative contribution of microbial versus plant-tissue metabolic pathways to ethanol removal; isotope-labelling or microbiome-exclusion experiments would help disentangle these mechanisms. Finally, whether the stomatal narrowing observed after prolonged exposure eventually limits removal capacity over multi-week or multi-month deployments was not tested and represents an important direction for future work, alongside evaluating performance under combined, realistic VOC mixtures rather than ethanol alone.

## 4. Materials and Methods

In the present study, healthy, broad-leaved *F. elastica* plants, with a minimum age of one year, were utilised. These plants were procured from a greenhouse in Türkiye. The primary rationale for the utilisation of *F. elastica* plants is their pervasive presence in both office and residential settings. These plants possess a substantial leaf surface area, exhibit remarkable adaptability to diverse environmental conditions, possess a high density of stomata on their leaf surface, and are endowed with a substantial cuticle layer.

### 4.1. Experimental Setup

**Figure 10:**
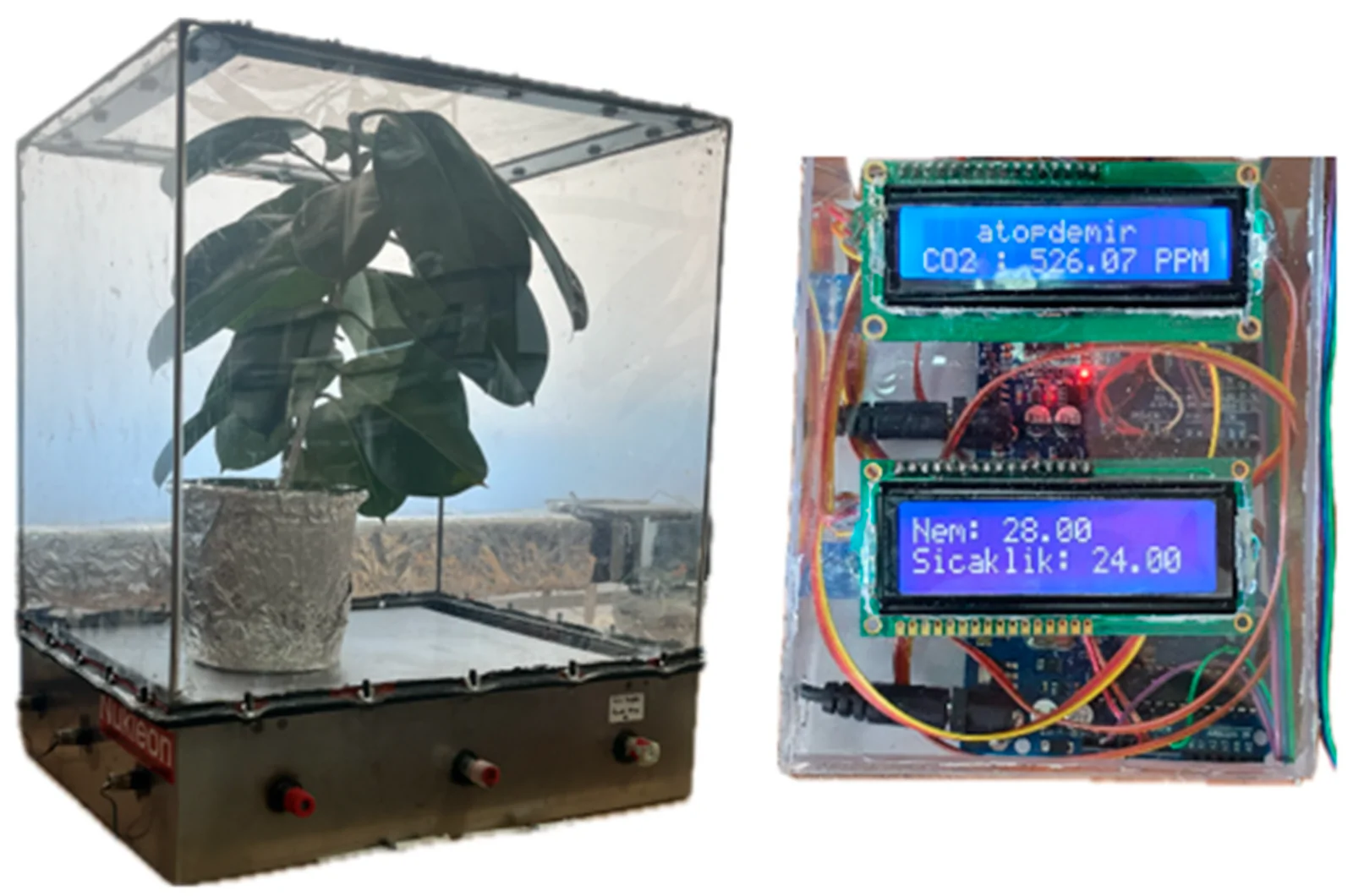
Test chamber used in experiments and monitor displaying real-time CO_2_ (ppm), relative humidity (%) and temperature (°C) values.

The test chamber (Figure 10) was designed to provide a controlled atmosphere with specific conditions, with the objective of optimising and observing the removal of VOCs from indoor air by the *F. elastica* plant. Experiments on environmental adaptation, VOC removal optimisation, desorption, and long-term VOC exposure were conducted in these chambers.

The test chamber is composed of two primary sections. The upper section is constituted of a transparent chamber made of 1 mm thick plexiglass, with dimensions of 65 × 50 × 60 cm, which facilitates observation without the presence of inlet or outlet ports. The lower section is composed of stainless steel, with dimensions of 15 × 50 × 65 cm, and contains the sensors, resistors, and fan. The section is equipped with a total of 12 inlet/outlet ports, three on each side, which are sealed with septum caps to ensure airtightness. A stainless steel plate measuring 55 × 49 cm is positioned between the upper and lower sections to accommodate the pot. A 5 mm gap is maintained on both sides of the plate to facilitate air circulation.

The contact points of the two sections forming the chamber are insulated with neoprene sealing tape to ensure gas tightness. Furthermore, a total of 20 screws, each measuring 2 cm in length, are tightened on the joint surfaces with the objective of preventing gas exchange between the system and the external environment. As part of the lighting system, 16 LED lamps are installed inside the chamber. The system operates on an automatic photoperiod schedule, providing 16 h of light and 8 h of darkness.

In this test chamber system, sensors were employed to control the internal environmental air parameters. These sensors were programmed and calibrated using two Arduino Uno boards. The MHZ-19 sensor was selected for the purpose of determining the carbon dioxide (CO_2_) concentration, whilst the relative humidity and temperature of the environment were monitored in real-time via DHT-11 sensors. A thermostat-controlled heating system has been integrated into the chamber, with the objective of automatically activating in response to sudden temperature drops.

Furthermore, a fan measuring 12 × 12 cm that is capable of continuous operation has been utilised to ensure uninterrupted air circulation within the confined space. The data obtained from the sensors was concurrently transferred to LED screens, thereby facilitating real-time monitoring. Within the scope of the lighting system, 16 CATA-CT2475 model LED lamps (Arslanlar, İstanbul, Türkiye) (36 W, 6400 K) were utilised. In order to ensure a constant CO_2_ concentration was maintained inside the chamber during the experiment, a cylinder containing 98% purity industrial-grade CO_2_ was utilised.

Air samples, with a volume of 1 cm^3^, were extracted from the test chamber’s internal environment to facilitate observation of alterations in concentration. Gas chromatography (Shimadzu GC-2010 Plus, Kyoto, Japan) (GC) was utilised to calculate concentration changes in the internal environment air during empty chamber tests, preliminary experiments, and optimisation experiments. The concentration changes in VOCs in the internal environment air were calculated using a gas chromatograph equipped with an Rtx-5MS (SGE-Trajan, Melbourne, Victoria, Australia) phase column (60 m, 0.32 mm, 0.5 μm) connected to a flame ionisation detector (FID). The carrier gas (nitrogen) flow rate in the column was set to 0.6 mL/min, the column temperature to 150 °C, the detector temperature to 200 °C, and the injection block temperature to 170 °C.

### 4.2. The Optimisation of Ethanol Removal

The optimisation of experimental data was achieved through the utilisation of Design Expert 10 software (v.10.0.4). The Response Surface Methodology (RSM) was utilised as the optimisation method. A series of experiments involving three variables—ethanol concentration (100–4000 ppm), relative humidity (40–90%), and duration (6–48 h)—was generated by the programme to optimise ethanol removal from a closed environment using the *F. elastica* plant. The experimental series obtained consisted of 20 different experiments within the relevant variable limits. The ethanol concentration range (100–4000 ppm) was deliberately selected to provide a comprehensive evaluation of the botanical filtration system across a broad range of operating conditions. Although the occupational exposure limit for ethanol is commonly established at approximately 1000 ppm as an 8 h time-weighted average, this value represents a regulatory exposure guideline rather than an experimental upper boundary. Concentrations exceeding this threshold may occur temporarily in poorly ventilated industrial environments, laboratories, healthcare facilities, and during accidental spills or intensive use of ethanol-based products, representing high-loading or worst-case exposure scenarios. Furthermore, the use of an extended concentration range was essential for the development of a robust RSM model, allowing reliable prediction of non-linear responses, identification of interactions among operating variables, and assessment of the maximum removal capacity and physiological tolerance of *F. elastica* under elevated ethanol loads.

The following equation (Equation (1)) was utilised for the conversion of VOC concentrations in the air (at 25 °C, 1 atm) from ppm to mg/m^3^. The conversion between these units was performed using the ideal gas relationship:

(1) mg/m³=(ppm)×(Molecular Weight)/24.45

The constant 24.45 represents the molar volume (L mol^−1^) of an ideal gas at 25 °C and 1 atm, which are the standard environmental conditions commonly adopted in indoor air quality studies.

The response variable utilised in this study was ethanol removal efficiency (RE), which is mathematically defined below (Equation (2)).

(2) RE=(1−Cx/C0)×100

In Equation (2), RE denotes removal efficiency (%), while C_0_ and Cx represent the initial and final VOC concentrations (ppm) in the chamber, respectively. All experiments were conducted at a light intensity of 135 µmol·m^−2^·s^−1^, a CO_2_ concentration of 500 ± 10 ppm, atmospheric pressure (1 atm), and a temperature of 25 °C. The temperature of the chamber was subject to regulation in order to counteract any temperature fluctuations that might have occurred externally. This was achieved by means of a resistive heater.

### 4.3. Observations of Post-Removal Desorption

Subsequent to the completion of the optimisation studies, the optimal environmental conditions that maximised removal efficiency were ascertained, and the plants were placed within the chamber. Ethanol removal experiments were conducted at initial concentrations of 1000 ppm for 48 and 72 h. In the 48 h experiment, injections equivalent to the initial ethanol concentration were performed at 0 and 24 h; in the 72 h experiment, injections were performed at 0, 24, and 48 h.

Subsequent to the termination of the specified periods, the plants were transferred to a second empty chamber that was of equivalent volume and technical characteristics, thereby minimising their contact with the external environment. Subsequent to the transfer, samples were collected from the closed environment air at 0, 15, 30, 60, 90, and 120 min, as well as at 5, 8, 12, 18, 24, 48, and 72 h. The air samples obtained were analysed using a gas chromatograph (GC) device to monitor changes in ethanol concentration in the internal environment. This facilitated the estimation of the concentration of ethanol released back into the environment from the plant subsequent to its removal of ethanol from the first chamber and subsequent transfer to an environment uncontaminated by any pollutants.

### 4.4. Long-Term Ethanol Exposure Test

A series of long-term exposure tests to ethanol were conducted in order to observe the effects of ethanol on commercially purchased plants. In order to achieve this objective, the leaf surfaces and pots of the plants were meticulously cleansed, and the plants were irrigated 48 h prior to the commencement of the experiment.

Plants subjected to the experiment were exposed to 2000 ppm ethanol for 120 h and maintained under photoperiods of 16 h of light and 8 h of darkness throughout the experiment. The CO_2_ concentration within the chamber was maintained at 500 ± 10 ppm, and the relative humidity was kept between 65 and 70%. Concurrently, as the ethanol concentration decreased, the frequency of ethanol injections was adjusted to a 24 h interval at the initial concentration level.

### 4.5. Analysis of the Amount of Bioactive Compounds in Leaf Explants

The analyses performed employed ethanol (Merck, Darmstadt, Germany), distilled water and ethyl acetate (BDH, GPR grade, Poole, England) as solvents. An acetone-water (*v*/*v*) solution with a concentration of 80% was utilised for the estimation of the total chlorophyll and carotenoid content of the leaf extracts. The results for total phenolic content, total flavonoid content, and total antioxidant capacity were obtained by substituting the relevant absorbance values into equations derived from a calibration curve created using specific gallic acid, quercetin, and Trolox concentrations.

The Folin–Ciocalteu method, as reported by Singleton and Rossi [47], was utilised to ascertain the total phenolic content. For the analyses, 1.5 mL of Folin–Ciocalteu reagent (Sigma-Aldrich, St. Louis, MO, USA) diluted 10-fold was mixed with 300 µL of *F. elastica* extract. Following a waiting period of approximately two minutes, 1.2 mL of a 7.5% Na_2_CO_3_ (Merck, Darmstadt, Germany) solution was added and thoroughly mixed. Subsequently, the absorbances of the mixtures, which had been stored in the dark for a period of 90 min, were measured in comparison to distilled water at a wavelength of 765 nm.

The spectrophotometric AlCl_3_ method was applied to determine the total flavonoid content in *F. elastica* extracts [48]. During the preparation of a 2% AlCl_3_ (Sigma-Aldrich, St. Louis, MO, USA) solution, glacial acetic acid and methanol (50%, *v*/*v*) were employed as the solvent. In this analysis, 1 mL of *F. elastica* extract and 1 mL of a 2% AlCl_3_ solution were mixed and left at room temperature for 10 min. Subsequently, the absorbances of the samples were measured against the blank (2% AlCl_3_) at a wavelength of 394 nm.

The total antioxidant capacities of the samples were evaluated using a method based on the scavenging ability of the ABTS (2,2′-azobis(3-ethylbenzothiazoline-6-sulfonate)) radical cation [49]. In this method, an ABTS (Thermo Fisher Scientific, Waltham, MA, USA) stock solution (7 mM) was prepared and subsequently diluted with phosphate buffer until its absorbance was approximately 0.7. The solution was then subjected to a stringent protocol to minimise exposure to light. Prior to the measurement of the absorbances, a mixture of 100 µL of *F. elastica* extract and 1900 µL of ABTS solution was prepared and incubated at room temperature. The absorbance values were measured at the end of the sixth minute and then compared to a phosphate buffer standard. The measurement was taken at a wavelength of 734 nm.

For the purpose of analysis, dried *F. elastica* leaves were ground into a powder using a grinder, thus allowing for the subsequent analysis of total chlorophyll and total carotenoid amounts. In this analysis, the concentration of the samples (mg/g) was calculated using the method reported by Arnon [50]. Subsequently, the absorbances measured at the wavelengths specified in the method were entered into the equations provided by Lichtenthaler and Wellburn [51]. These equations were then solved to calculate the relevant parameters.

Scanning electron microscope (SEM) analyses were performed at a magnification of 2.00 KX using a Zeiss EVO-MA10 (Zeiss, Oberkochen, Germany) SEM to observe the changes after optimisation and long-term exposure processes for the leaf and root parts of the plant to be used in the removal of ethanol. Samples of *F. elastica* were collected from each experimental group and cut into small sections. These were then dried in a laboratory oven (Memmert, Schwabach, Germany) at 55 °C for 48 h. The dried specimens were mounted on stubs using double-sided conductive carbon tape and sputter-coated with a thin layer of gold to improve surface conductivity. No chemical fixation or graded dehydration procedures were applied in this study.

Artificial intelligence (AI) tools were used exclusively for literature exploration, language translation, text editing, and grammatical improvement during the preparation of this manuscript. All scientific content, experimental design, data analysis, results, figures, tables, and conclusions were developed and verified solely by the authors. No results, figures, graphs, or other scientific outputs presented in this study were generated by AI.

## 5. Conclusions

In this study, ethanol removal from indoor air using *F. elastica* Roxb. was successfully optimised, and system performance was validated through both statistical modelling and experimental verification. Response Surface Methodology predicted a maximum removal efficiency of 97.16% under optimal conditions of approximately 2280 ppm initial ethanol concentration, ~67% relative humidity, and ~41 h of exposure time. Verification experiments yielded an average removal efficiency of 96.2%, confirming the high predictive accuracy and reliability of the developed model. ANOVA results demonstrated that exposure time was the most influential parameter affecting ethanol removal, while initial concentration and relative humidity exerted secondary but statistically significant effects. The high removal performance observed in this study suggests that ethanol removal involved both physical uptake and biological transformation within the plant, as supported by the limited desorption observed following pollutant removal.

Desorption experiments indicated that although limited re-emission of ethanol occurred after pollutant removal, the released concentrations were low and transient. These findings suggest that ethanol was not merely physically adsorbed but was at least partially metabolised by plant tissues and/or degraded by associated microorganisms. Scanning electron microscopy analyses revealed partial deformation and narrowing of stomatal structures, along with alterations in the cuticular layer following prolonged ethanol exposure. Concurrent increases in total phenolic content, flavonoid concentration, and antioxidant capacity further indicate activation of plant defence mechanisms under ethanol-induced stress. However, the observed reduction in total chlorophyll content under long-term high-concentration exposure suggests potential constraints on photosynthetic performance at elevated pollutant levels.

Overall, *F. elastica* demonstrated considerable potential as a botanical filtration system for ethanol removal under controlled experimental conditions. Optimisation of environmental parameters significantly improved removal efficiency, while physiological and biochemical analyses provided valuable insights into the plant’s response to prolonged ethanol exposure. However, the present study was conducted under sealed chamber conditions using relatively high ethanol concentrations and evaluated only the short-term response of a single plant species. Therefore, caution should be exercised when extrapolating these findings to real indoor environments. Future studies should investigate the long-term performance of botanical filtration systems under realistic indoor VOC concentrations, dynamic ventilation conditions, and multiple plant species to better assess their practical applicability. Also, future research should focus on detailed characterisation of rhizospheric microbial communities and quantitative assessment of their contribution to VOC degradation, which would further strengthen the scalability and industrial applicability of plant-based biofiltration technologies.

## Figures and Tables

**Table 1:** Percentage losses were measured in the test chamber at the end of 48 h. Data are expressed as mean ± standard deviation (SD), based on three independent replicates (*n* = 3).

	Test Chamber	Empty Pot	Potted Test Plant
Total Loss, at 48 h (%)	32.7 ± 0.8	39.4 ± 0.9	82.0 ± 1.2

**Table 2:** ANOVA table for ethanol removal optimisation.

Source	Sum of Squares	df	Mean Square	F Value	*p*-Value
Model	2859.85	9	317.76	330.81	<0.0001
A—Ethanol Concentration	68.86	1	68.86	71.69	<0.0001
B—Relative Humidity	16.96	1	16.96	17.66	0.0018
C—Time	2121.90	1	2121.90	2209.07	<0.0001
AB	0.24	1	0.24	0.26	0.6245
AC	2.64	1	2.64	2.75	0.1280
BC	2.00	1	2.00	2.08	0.1796
A^2^	228.20	1	228.20	237.58	<0.0001
B^2^	76.94	1	76.94	80.10	<0.0001
C^2^	445.46	1	445.46	463.76	<0.0001
Residual	9.61	10	0.96		
Lack of Fit	9.61	5	1.92	—	<0.0001
Total	2869.46	19			

**Table 3:** Model fit and statistical summary.

Statistical Parameter	Value
Standard Deviation (Std. Dev.)	0.98
Mean	81.89
Coefficient of Variation (C.V. %)	1.20
PRESS (Predicted Residual Error Sum of Squares)	73.88
−2 Log Likelihood	42.09
R-Squared (R^2^)	0.9967
Adjusted R-Squared (Adj R^2^)	0.9936
Predicted R-Squared (Pred R^2^)	0.9743
Adequate Precision	60.499
BIC (Bayesian Information Criterion)	72.05
AICc (Corrected Akaike Information Criterion)	86.53

**Table 4:** Solutions presented for the maximum ethanol removal percentage response variable using RSM optimisation within the specified variable value ranges.

Solution	Ethanol Concentration (ppm)	Relative Humidity (%)	Time (h)	Ethanol Removal Efficiency (%)	Desirability
1	2282.089	66.705	40.809	97.160	0.941
2	2281.516	66.655	40.804	97.160	0.941
3	2282.120	66.760	40.823	97.160	0.941
4	2276.239	66.696	40.806	97.160	0.941

**Table 5:** The mean result obtained from a series of five validation tests, conducted under the specified conditions for the purpose of determining maximum ethanol removal, was 97.160%.

No.	Ethanol Concentration (ppm)	Relative Humidity (%)	Time (h)	Ethanol Removal Efficiency (%)
1	2282.0	67.0	40.8	95.8
2	2282.0	67.0	40.8	96.5
3	2282.0	67.0	40.8	96.3
4	2282.0	67.0	40.8	95.7
5	2282.0	67.0	40.8	96.7
Mean	–	–	–	96.2

**Table 6:** Ethanol removal efficiency across ten consecutive trials performed on the same micropropagated *F. elastica* test unit (two co-cultivated plants in a single pot, total leaf area = 0.81 m^2^).

Experiment No.	Ethanol Concentration (ppm)	Relative Humidity (%)	Time (h)	Ethanol Removal Efficiency (%)	Leaf Surface Area (m^2^)	Removal per Unit Surface Area (ppm/m^2^)
1	2282.0	67.0	40.8	93.5	0.81	2634.26
2	2282.0	67.0	40.8	94.0	0.81	2648.35
3	2282.0	67.0	40.8	94.4	0.81	2659.62
4	2282.0	67.0	40.8	94.7	0.81	2668.07
5	2282.0	67.0	40.8	94.9	0.81	2673.71
6	2282.0	67.0	40.8	95.1	0.81	2679.35
7	2282.0	67.0	40.8	95.3	0.81	2684.99
8	2282.0	67.0	40.8	95.8	0.81	2699.08
9	2282.0	67.0	40.8	95.9	0.81	2701.90
10	2282.0	67.0	40.8	95.6	0.81	2693.45

**Table 7:** Total phenolic compound content of leaf extracts obtained using different solvents (mg/g GAE) (mean ± standard deviation, n = 3).

Solvent	120 E	Unexposed Plant
Distilled Water	21.28 ± 1.65	17.43 ± 1.08
Ethanol	48.38 ± 2.04	43.78 ± 1.89
Ethyl Acetate	58.14 ± 2.44	52.98 ± 2.04

**Table 8:** Total flavonoid content of leaf extracts obtained using different solvents (mg/g QE) (mean ± standard deviation, n = 3).

Solvent	120 E	Unexposed Plant
Distilled Water	26.63 ± 1.48	25.28 ± 1.83
Ethanol	50.53 ± 1.84	46.17 ± 2.15
Ethyl Acetate	64.38 ± 2.97	60.06 ± 3.13

**Table 9:** Total antioxidant capacity of leaf extracts obtained using different solvents (mmol/g TEAC) (mean ± standard deviation, n = 3).

Solvent	120 E	Unexposed Plant
Distilled Water	8.09 ± 0.33	7.85 ± 0.20
Ethanol	8.24 ± 0.29	7.27 ± 0.19
Ethyl Acetate	9.23 ± 0.42	8.83 ± 0.37

**Table 10:** The total chlorophyll, chlorophyll-a, chlorophyll-b and total carotenoid content (mg/g dry leaf) obtained from *F. elastica* leaf extracts (mean ± standard deviation, n = 3).

Parameter	120 E	Unexposed Plant
Total Chlorophyll	1.12 ± 0.13	1.44 ± 0.14
Chlorophyll-a	0.72 ± 0.10	0.93 ± 0.14
Chlorophyll-b	0.21 ± 0.09	0.37 ± 0.11
Total Carotenoid	0.61 ± 0.16	0.54 ± 0.15

## Data Availability

The data supporting the findings of this study are available from the authors upon reasonable request.

## Abbreviations

The following abbreviations are used in this manuscript:

VOC	Volatile Organic Compounds
LED	Light Emitting Diode
GC	Gas Chromatography
RSM	Response Surface Methodology
RE	Removal Efficiency
ABTS	2,2′-azobis(3-ethylbenzothiazoline-6-sulfonate)
SEM	Scanning Electron Microscope
GAE	Gallic Acid Equivalents
QE	Quercetin Equivalents
TEAC	Trolox Equivalent Antioxidant Capacity
ACGIH	American Conference of Governmental Industrial Hygienists
NIOSH	National Institute for Occupational Safety and Health
